# Atrioventricular Junction Ablation with High-Definition Recording of Atrioventricular Node Potential

**DOI:** 10.3390/jcdd12120479

**Published:** 2025-12-04

**Authors:** Andrea Matteucci, Enrico Maggio, Domenico Dardani, Maurizio Russo, Marco Galeazzi, Federico Nardi, Silvio Fedele, Claudio Pandozi, Furio Colivicchi

**Affiliations:** 1 Clinical and Rehabilitation Cardiology Division, San Filippo Neri Hospital, 00135 Rome, Italy; 2 Department of Experimental Medicine, Tor Vergata University, 00133 Rome, Italy; 3 Department of Cardiovascular, Respiratory, Nephrological, Anesthesiological and Geriatric Sciences, “Sapienza” University of Rome, 00185 Rome, Italy; 4 Department of Clinical and Molecular Medicine, Sapienza University of Rome, 00189 Rome, Italy; 5 Cardiology Unit, Santo Spirito Hospital, Casale Monferrato, 15033 Alessandria, Italy; 6 Department of Interventional Cardiology, UOC Cardiologia, Sandro Pertini Hospital, 00157 Rome, Italy

**Keywords:** atrioventricular node ablation, atrial fibrillation, tachycardiomyopathy, electroanatomical mapping, atrioventricular node potential

## Abstract

Atrioventricular (AV) node ablation represents an established therapeutic option in the management of atrial fibrillation (AF) and other atrial tachyarrhythmias, particularly in patients with symptomatic tachycardia who remain unresponsive or intolerant to pharmacological therapy. The procedure is often considered in cases of refractory arrhythmias, antiarrhythmic drugs intolerance, or tachycardiomyopathy, and plays a key role in optimizing outcomes in patients undergoing cardiac resynchronization therapy, where achieving adequate biventricular pacing is otherwise compromised by rapid ventricular responses. Traditionally, AV node ablation is performed using radiofrequency energy delivered at the region of the His bundle, guided by the earliest His potential recordings. However, the anatomical complexity of the AV node and Koch’s triangle poses important challenges, including the risk of incomplete ablation, persistence of conduction, lack of reliable junctional escape rhythms, and increased risk of proarrhythmia. Recent advances in high-resolution mapping and electroanatomical guidance have enabled a more precise anatomical approach, selectively targeting the compact AV node while reducing collateral injury. These developments offer the potential for improved procedural safety, long-term efficacy, and a more standardized strategy for patient management. This review summarizes current evidence, techniques, and clinical implications of AV node ablation, highlighting its role in the evolving landscape of arrhythmia treatment.

## 1. Introduction

The procedure of atrioventricular (AV) node ablation is useful in managing patients in several clinical scenarios. It is frequently chosen in patients with atrial fibrillation (AF) who experience symptomatic tachycardia and in those who are unresponsive to medical therapy, and is commonly indicated in patients with refractory atrial tachyarrhythmias, such as atypical atrial flutter with rapid ventricular response, where AV node ablation offers a solution when pharmacological or electrical interventions fail to maintain rate control. It is also particularly beneficial in individuals who are intolerant to antiarrhythmic medications. AV node ablation can be employed in patients who undergo pacemaker implantation, allowing for a more stable and controlled conduction system and improving resynchronization therapy when the target of biventricular stimulation of 90% of the time cannot be achieved due to the rapid ventricular response despite pharmacological treatment. Finally, it can be resolutive in case of tachycardiomyopathy [1,2,3]. When an AV node ablation is performed, it should be coupled with pacemaker implantation. The classic technique consists of the delivery of radiofrequencies at the site where an atrial deflection with the earliest His bundle potential, followed by the ventricular deflection from the right or left septum, is recorded. Another approach targets the site with the most prominent His bundle potential. In both techniques, in the case of AF, the His bundle potential is the only feature guiding the chosen site of ablation [4]. An important challenge is the recurrence of tachycardia, which may be attributed to the unique anatomy of the AV node and Koch’s triangle [5,6]. This approach does not guarantee disruption of the compact AV node itself, often sparing the nodal tissue and instead injuring the distal non-branching His bundle. Such lesions may result in suboptimal procedural outcomes, including a lack of a reliable junctional escape rhythm and a higher risk for ventricular arrhythmias. Recent evidence has suggested that a more anatomically grounded ablation strategy, guided by high-definition recording techniques and structural mapping, can selectively and effectively target the compact AV node. This review explores the rationale, methodology, and clinical implications of anatomical AV node ablation using high-resolution electroanatomical guidance.

## 2. Properties and Characteristics of the AV Node

**Figure 1:**
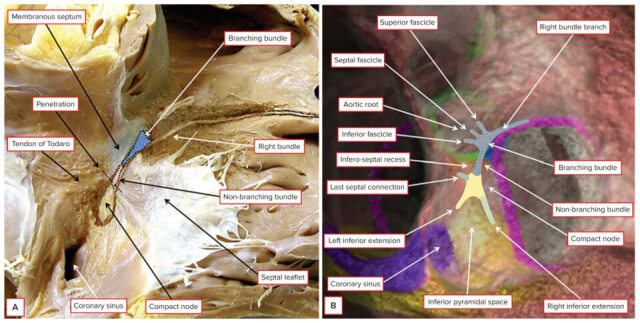
Panel (**A**): Dissection of the heart tissue reveals the distinct texture differences between specialized and functional myocardium, allowing for an approximate identification of the atrioventricular conduction axis components. The extensions of the nodal structures are indicated by dashed lines superimposed on the dissection. Panel (**B**) illustrates the application of the conduction axis onto a living dataset, based on prior studies where the axis was successfully mapped in autopsied hearts through histological analysis. In this approach, the atrioventricular node and its extensions have been reconstructed within a segmented dataset of the atrioventricular and ventriculo-arterial junctions using CT imaging. The His potential is recorded at the location of the non-branching bundle. Reproduced from [4].

Traditionally, the area known as Koch’s triangle is identified by the fibrous tendon of Todaro, the tricuspid valve leaflet attachment, the ostium of the coronary sinus, and the septal isthmus; nevertheless, more recent evidence proposed the new definition of Koch’s pyramid, due to its relationship with the pyramid space and inferoseptal recess [4,7]. The AV node (also known as the node of Aschoff-Tawara) has a length of 5–7 mm and a width of 1.0–1.5 mm. The non-branching component of the conduction axis was shown to be 3.6 ± 1.7 mm in length, varying from 1.7 to 7.2 mm. The AV node can then be divided into the lower nodal bundle and the compact node. The distinction between the AV node and the non-branching bundle is made at the point where the conduction axis becomes insulated by the fibrous tissues of the AV junctions from the working atrial myocardium. The cells of the lower nodal bundle are longer and more parallel to one another, whereas in the compact node, they are smaller and without a clear orientation. In humans, we can find two AV nodal extensions: the rightward nodal extension goes proximally from the lower nodal bundle towards the coronary sinus. In contrast, the leftward nodal extension goes from the compact node towards the coronary sinus (and therefore is usually shorter than the other one) [4,8,9,10] (Figure 1).

Regarding the AV junction (AVJ), notable for compartmentalization of connexin Cx43 expression, it is formed by the AV node, rightward and leftward nodal extension (with their transitional cells), His bundle, and AV fibrous ring. It has the role of coordinating and maintaining appropriate AV conduction, protecting the ventricles from high rates due to atrial tachyarrhythmias, and functioning also as a backup pacemaker in the setting of sinoatrial node dysfunction. Considering its role, an abnormal impulse propagation in the AVJ can be associated with AV block and AV node re-entry tachycardia. In the latter, there is a reentrant circuit involving the AVJ functionally conducted through the fast and slow pathways. The gap junction protein Cx43 is the most representative connexin in the human heart. Its expression seems to be similar in rightward nodal extensions, lower nodal bundle, and His bundle, with approximately half of the interatrial septum and transitional cells expressing; this is higher than the Cx43 expression in leftward nodal extensions and compact node. This differential Cx43 expression in the different cell types of the AVJ, likely driven by different gene expression programs, may provide each structure with unique conduction properties, contributing to arrhythmias arising from this region. Other human heart connexins are Cx37, Cx40, Cx45, and Cx46 [11,12,13]. The atrial components of the conduction axis, located within the pyramid of Koch and therefore part of the septal vestibule of the tricuspid valve, are close to important heart structures, such as the mouth of the coronary sinus, the membranous septum and the septal isthmus, the inferior vena cava, and the aortic root with the aortic valve. It is important to have in mind these anatomical proximities to avoid complications during the AV node ablation procedure [14,15,16].

The vascularization for the AV node originated most frequently from the right coronary artery (90% of cases; usually in cases of right or balanced arterial coronary dominance) at the level of the crux cordis, while in the other 10% it arose as a branch of the left coronary artery. (usually in cases of left dominance). The AV node artery appears as the first, inferior, and longest septal perforating branch that always arises at the level of the crux cordis and goes into the cardiac wall towards the right fibrous trigone, near to the mitral valvular ring [17]. The main venous route from the AV node and the His bundle passes into the thebesian vein, which opens in roughly 80% of cases into the right atrium, next to the coronary sinus. Another route goes from the same structures via a vein that accompanies the AVN artery, draining eventually into the middle cardiac vein. The third route takes venous blood from the AVJ and is drained to the tributaries of the great cardiac vein, interconnecting with the branches of the above two veins. The venous return from the ventricular bundle-branches is drained into the oblique septal veins [18].

The AV node and sinoatrial node are more densely innervated than the His bundle and bundle branches, and the bundle branches receive more innervation than the ventricular myocardium. Therefore, it appears evident that the existence of a proximal-distal parasympathetic innervation gradient in the heart. With an opposite gradient, most of the sympathetic innervation is located in the ventricle (in the His bundle, the sympathetic component is already dominant) [19,20,21,22,23]. AV node cells generally have a higher density of β1- and β2-adrenergic and acetylcholine receptors. Catecholamine-mediated phosphorylation of L-type calcium channels enhances inward calcium currents, increasing AV node conduction velocity. Furthermore, catecholamines increase AV node automaticity, activating the current If with the diastolic release of calcium from the sarcoplasmic reticulum. Acetylcholine and adenosine exert potent negative dromotropic effects, reducing intracellular cAMP levels and leading to decreases in the transmembrane calcium current. They also activate the outward potassium current IK, which reduces the action potential duration. Nevertheless, these molecules prolong post-repolarization refractoriness, probably due to slowed recovery of ICa-L from inactivation. These combined effects slow AV node conduction velocity, promote decremental conduction, produce Wenckebach-type conduction block, and reduce automaticity. Interestingly, low doses of adenosine selectively affect the anterograde AV node fast pathway and therefore can unmask slow pathway conduction and induce AV node re-entry tachycardia [24].

The AV node controls heart synchrony, generating a delay between atrial and His bundle activation. This delay results from a conduction slower than in other myocardial tissues and accounts for approximately half of the normal PR interval. During conditions of high sympathetic activity, conduction accelerates through the AV node, maintaining optimal ventricular filling during faster heart rates. The delay generated by the AV node can also block some premature impulses, acting as a filter for fast atrial impulses, and therefore it prevents the occurrence of life-threatening ventricular tachyarrhythmias. Indeed, impulse propagation across the AV node is characterized by decremental conduction. Nevertheless, when the delay becomes too high or heterogeneous in the AV node, pathological conduction blocks or reentrant tachycardias may appear. Finally, the AV node can act as a subsidiary pacemaker in case of sinoatrial node disease. The AV node presents a fast pathway and a slow pathway, both able to transmit the atrial impulse to the His bundle. A premature atrial impulse may be blocked by the long refractory period of the fast pathway, but its conduction through the slow pathway may be long enough to allow the fast pathway to recover excitability and be activated in the reverse direction, leading to a reentrant atrial activation. Such reentry may take a variety of forms and lead to an AV node re-entry tachycardia [25,26,27]. Variability exists in the compact AV node location within the atrioventricular junction, affecting electrophysiological properties [8]. Indeed, we can identify a slow-fast conduction (90% of cases), a fast-slow conduction (8%), and a slow-slow conduction (2%) [28]. The typical fast pathway ablation sites are located anterosuperior toward the apex of the triangle of Koch, close to the compact AV node. Instead, the usual slow pathway ablation sites are located posteroinferior toward the base of the triangle of Koch, at a greater distance from the compact AV node and bundle of His. Variability also exists in the fascicles of the left bundle, which extend in the supero-lateral and infero-medial papillary muscles. The superior fascicle has a variable relationship to the attachment of the right coronary aortic valvar leaflet. This variability could be one of the determinants of the vulnerability of the conduction axis after transcatheter aortic valve replacement (TAVR). Instead, the portion of the conduction axis related to the base of the atrial septum is inferior to the aortic root, and therefore, it should not be at risk during the AVJ ablation procedure [29].

## 3. AV Node Ablation and Post-Ablation Proarrhythmic Risks

Histologically specialized areas of the vestibules of the tricuspid and mitral valves (which form the atrial walls of the pyramid of Koch) expand to become the compact AV node. This area receives direct connections from the working myocardium of the atrial septum. This area appears to be isolated by the fibrous tissue of the AVJ, and the axis becomes the non-branching AV bundle (His bundle). Therefore, the recording made at the site after its insulation by the fibrous tissues of the AVJ will show the His bundle activation along with atrial and ventricular signals. The AV node itself, however, is positioned just inferior to the site of insulation of the conduction axis [4]. In traditional AV junction ablation procedures—especially in AF patients where no organized atrial activity is available—the location for RF delivery is determined solely by the recording of a prominent and early His potential. While this may indicate proximity to the insulated His bundle, it does not necessarily correlate with the position of the compact AV node. This discrepancy carries several risks. Firstly, the need for multiple RF applications may lead to excessive myocardial injury and formation of a large scar, which is potentially proarrhythmic. Secondly, ablation of the His bundle rather than the AV node often eliminates the intrinsic junctional escape mechanism, necessitating immediate and permanent pacing. Lastly, the abrupt cessation of AV conduction and rapid change in ventricular activation pattern has been associated with increased QT dispersion and vulnerability to torsades de pointes, particularly in patients with underlying structural heart disease.

**Figure 2:**
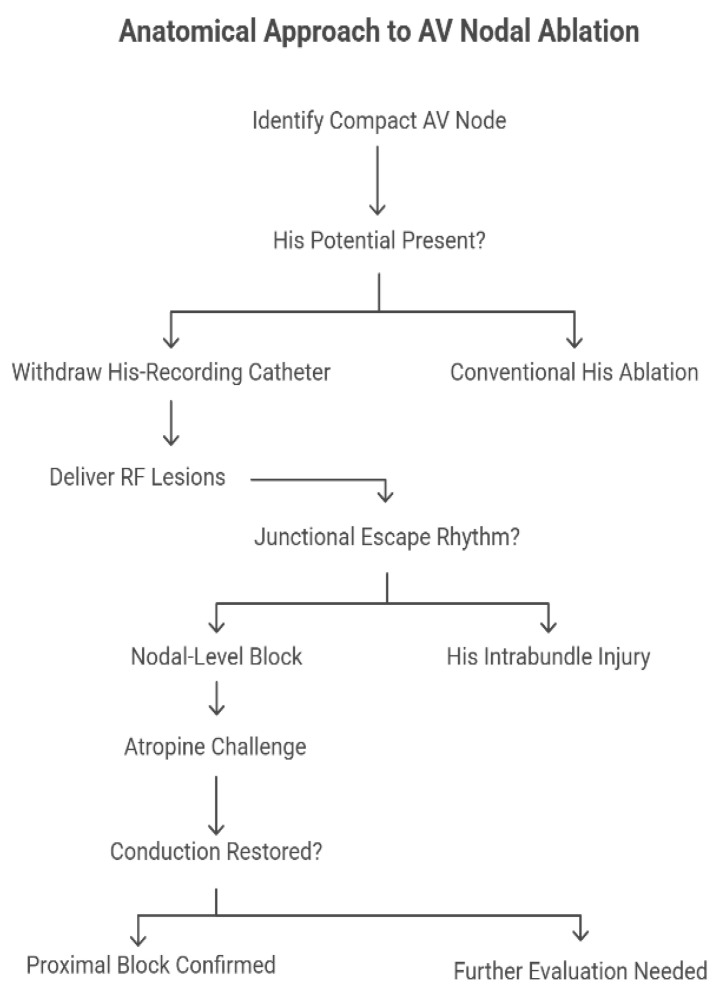
Anatomical approach to AV nodal ablation. Schematic representation of the anatomical technique for AV junction ablation targeting the compact atrioventricular node rather than the insulated His bundle. After identification of the compact AV node within the Koch’s triangle, the operator determines whether a His potential is present. In the conventional His ablation approach (right branch), RF energy is delivered at the site of a prominent His potential, often resulting in His bundle injury and loss of intrinsic junctional escape rhythm. Conversely, in the anatomical approach (left branch), the His-recording catheter is gradually withdrawn until the His potential disappears, marking the probable location of the compact AV node. Radiofrequency lesions are then applied at this level to achieve nodal-level block while preserving a stable junctional escape rhythm. The subsequent atropine challenge confirms whether conduction can be restored, thereby distinguishing a proximal nodal block from an incomplete lesion requiring further evaluation.

Responding to these concerns, a targeted anatomical strategy has been proposed and recently evaluated, whereby the compact AV node is selectively ablated based on anatomical landmarks rather than electrophysiological potentials alone. Gross dissective studies, histological sections, and three-dimensional reconstructions (including CT datasets) have precisely located the compact node just inferior and posterior to the site of His bundle penetration within the pyramid of Koch. The mapping technique consists of withdrawing the His-recording catheter until the His potential disappears, isolating the approximation of the compact node before delivering RF lesions (Figure 2).

Katritsis et al. [4] reported that this technique achieved acute AV nodal block in 87.5% of a cohort of 72 patients with a median procedural time of 60 min, median fluoroscopy exposure of 3.4 min, and a median of 4 RF lesions. Notably, in 71% of successful cases, a junctional escape rhythm emerged—with a narrow QRS matching baseline morphology—suggesting true nodal-level block rather than His intrabundle injury. Importantly, atropine challenge in six patients did not restore conduction, reinforcing the proximal location of the block.

For the small proportion of patients in whom this anatomical approach failed, the protocol converged to a standard His-bundle ablation, requiring significantly more lesions (median 12) and failing to produce a junctional escape rhythm in all cases, which underscores the potential advantage of the anatomical approach, not merely effectiveness but also sparing of distal conduction tissues to preserve a physiologic escape rhythm.

The safety profile was reassuring: over a median follow-up of 10.5 months, persistent AV block was maintained in all cases, with no reported sudden deaths or sustained ventricular arrhythmias. Despite its benefits, the anatomical approach to AV nodal ablation is not universally applicable. In patients with persistent AF where no distinct His potential can be recorded, anatomical localization is less precise, limiting the utility of the withdrawal technique. In these cases, operators may be forced to default to conventional His ablation. Furthermore, variability in nodal anatomy and insulation patterns within the triangle of Koch may impact procedural success. Theoretically, the use of irrigated RF catheters or contact force-sensing systems could improve lesion formation, but these technologies were not uniformly employed, and their specific contribution remains unclear.

## 4. High-Definition Recording of AV Node Potential in AV Node Ablation

**Figure 3:**
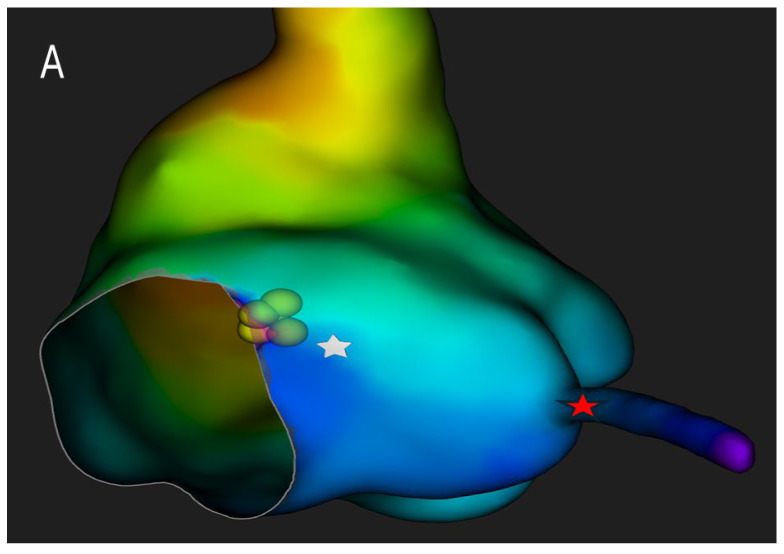

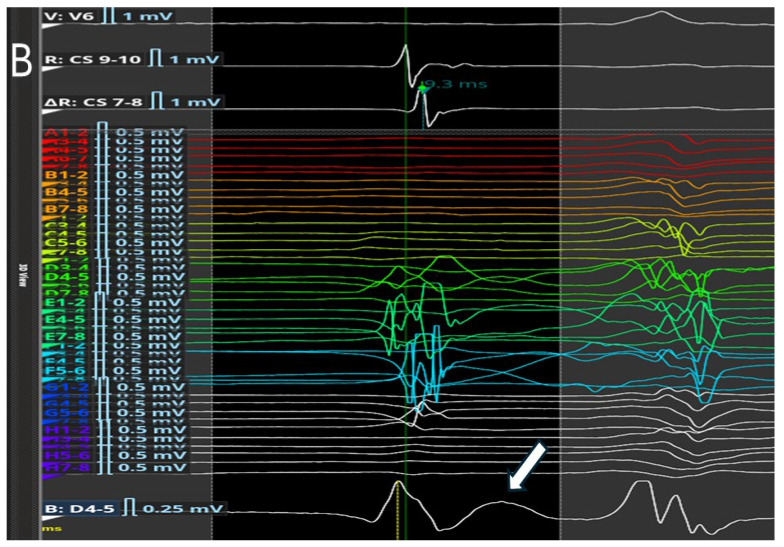
Panel (**A**). Three-dimensional electroanatomical reconstruction of Koch’s triangle showing the spatial localization of the compact atrioventricular node (white star) immediately inferior to the His bundle region. The red star indicates the coronary sinus. Panel (**B**). High-fidelity intracardiac recording obtained with a high-density mapping catheter using bipolar filtering (0.1–250 Hz). The electrogram at the ablation site (bottom trace) exhibits the characteristic features of the compact AV nodal potential: a long-duration, low-frequency, low-amplitude “hump” deflection occurring between the atrial and His potentials (white arrow). This signal, confined to the mid- and posteroseptal portions of Koch’s triangle, precedes the onset of the His deflection and disappears outside this region, confirming its origin from the compact AV node. The simultaneous color-coded activation map demonstrates the anatomical relationship between the nodal electrogram and the surrounding structures, guiding precise lesion delivery while preserving distal conduction.

High-resolution mapping systems and specialized filtering settings, such as those used in our previous studies [30,31], may improve visualization of nodal potentials and enhance procedural precision. Using a catheter with small electrodes closely spaced from each other (0.4 mm2 area and 2.5 mm spacing) and an unconventional filtering of the bipolar electrogram (0.1–250) with high signal amplification, it is possible to appreciate the characteristics of AV node potential during both sinus rhythm and slow-fast AV node re-entry tachycardia. These characteristics are: (1) long—duration, low—frequency, low—amplitude AV node hump signal recorded by the electrodes overlying the AV node; (2) markable in the middle of AV node signal duration when no His activation is detected in the far field or at the beginning of His-like activation when this last is simultaneously recorded; (3) impossibility of recording this slow potential outside the Koch’s triangle; (4) recordable only in the mid—septal and posteroseptal regions of Koch’s triangle; (5) not observable after the blocked atrial electrogram during the Wenckebach sequences. Interestingly, the AV node potential had a significantly shorter duration and greater amplitude in sinus rhythm than in tachycardia, while during atrial stimulation, there was a widening and a decrease in the amplitude of the AV node potential. Furthermore, the AV node potential began before the high-frequency His deflection and ended after its offset during sinus rhythm, whereas during tachycardia, the AV node potential always preceded the onset of His deflection in the near field. By recording potentials in the posteroseptal region of Koch’s triangle (slow pathway region), it is possible to appreciate the AV node potential (low frequency and low amplitude) together with a high-frequency slow pathway Jackman potential, located just before the onset of the AV node potential (Figure 3).

**Figure 4:**
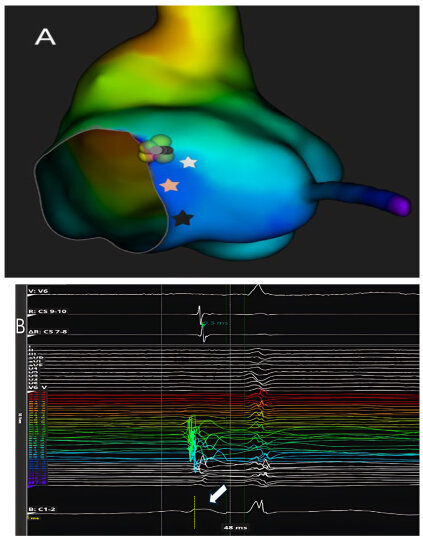

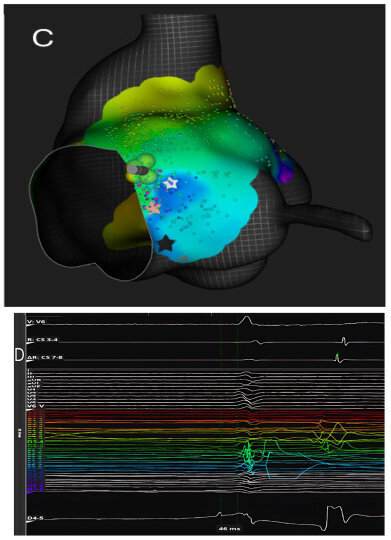
High-definition electroanatomical mapping of the compact AV node before (Panel (**A**)) and after ablation (Panel (**C**)). Three-dimensional high-resolution electroanatomical maps (Panels (**A**,**C**)) and corresponding intracardiac electrograms (Panels (**B**,**D**)) acquired before (Panel (**B**)) and after (Panel (**D**)) AV nodal ablation. The compact atrioventricular node (white star) was identified within Koch’s triangle by means of high-density mapping and bipolar filtering (0.1–250 Hz), displaying a characteristic long-duration, low-amplitude, low-frequency “hump” potential (white arrow) confined to the mid- and posteroseptal regions. This nodal potential preceded the onset of the His deflection during sinus rhythm, confirming its origin from the compact AV node. The color-coded activation map illustrates the spatial distribution of nodal and His signals, with ablation lesions (colored spheres) delivered at the anatomical level corresponding to the compact node. Post-ablation recordings demonstrate persistence of a normal His-ventricular interval, indicating preservation of distal conduction through the His–Purkinje system and confirming the selective nature of the nodal lesion.

These recordings can also distinguish between the slow potential originating from the SP and the slow potential originating from the compact AV node; in fact, successful ablation has often been achieved at sites where an SP-AV node potential was recorded, without any AV block occurring. Therefore, proceeding in order from the SP region towards the His region, the following can be recorded: (1) the AV node potential together with the SP potential; (2) AV node potential alone; (3) AV node potential hump and high-frequency His potential; (4) His potential alone (Figure 4).

**Figure 5:**
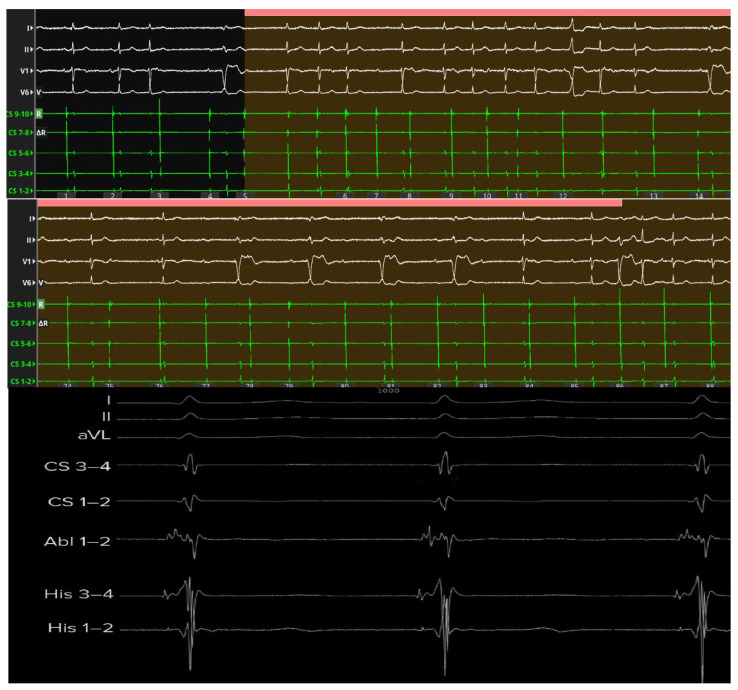
Junctional escape rhythm at the end of radiofrequency delivery. Junctional escape rhythm appearing immediately after cessation of radiofrequency (RF) energy delivery at the anatomical site of the atrioventricular node, spatially distinct from the His bundle. The ablation catheter records an atrial electrogram occurring later than that recorded by both right- and left-sided His bundle catheters, confirming its nodal—not Hisian—location. The emergence of a stable junctional rhythm following energy delivery indicates successful nodal-level block with preservation of a physiological escape focus. Abbreviations: Abl = ablation catheter; CS = coronary sinus; His = His bundle recording.

Beyond technical precision, the ability to localize the compact AV node and its extensions carries major clinical implications. Targeted ablation within the defined nodal zone reduces the risk of His bundle injury, preserves intrinsic junctional escape rhythm, and minimizes the occurrence of proarrhythmic scar formation or QRS widening (Figure 5).

The fusion of HD mapping with three-dimensional anatomical reconstruction—optionally enhanced by periprocedural CT or MR integration—represents a paradigm shift from empirical lesion placement to precision-guided substrate modification. Future investigations should expand on how electroanatomical visualization of nodal structures may refine ablation endpoints, improve procedural safety, and elucidate the dynamic relationship between the compact AV node, its inputs, and the His bundle during physiological and arrhythmic conduction.

## 5. Future Perspectives

In light of the above, it is clear that transcatheter ablation procedures can be made more accurate and safer through a more accurate assessment of the anatomical structures responsible for arrhythmia and their relationship with adjacent structures. This can be achieved through various strategies, such as a more in-depth study of the potentials obtained from high-density three-dimensional electroanatomical mapping, or its integration with cardiac imaging methods such as CT or MR. Probably, the strategy that would allow the highest ablation success rate and, at the same time, the lowest rate of iatrogenic complications is the integration of cardiac imaging and high-intensity electroanatomical mapping. However, this may not be feasible in clinical practice. In fact, several problems can be encountered in real life. Firstly, cardiac CT and MR require modern equipment, which is not always available in all centers, especially in more peripheral ones. Similarly, these methods require highly specialized personnel to reduce false positive and false negative rates. Furthermore, not all healthcare systems may be able to accommodate and manage such an increase in demand for these services, especially in terms of cost perspective. Another important point to note is the standardization of the proposed approach. Indeed, to further improve the procedure, it would be appropriate to conduct external validation on a larger patient cohort and to develop local protocols to adapt the method to individual workplaces, while maintaining similar efficacy and safety parameters. Furthermore, for this promising method to become widely used, it would be necessary to have an operator who is already experienced in this method and able to train other colleagues for the few sessions needed to learn the approach, which is relatively simple (or have the necessary resources to pay for an experienced proctor). Moreover, it would be necessary to have the resources to cover the cost of the catheter and any other additional costs. To make this feasible, it would be advisable to carry out studies and cost-effectiveness assessments to confirm the validity of the method from a health economics perspective. Finally, it would be desirable for manufacturers to develop increasingly accurate high-definition mapping catheters to make the procedure faster, safer, and more effective. In our opinion, it would therefore be appropriate for future studies, in addition to continuing the validation of these integrated imaging methods and the development of others aimed at further optimizing catheter ablation in terms of efficacy and safety, to also consider continuing research into cost reduction and improving patient care, to better guarantee high standards of quality, efficacy, and safety.

## 6. Conclusions

Anatomical ablation of the compact AV node, guided by high-definition electro-anatomical mapping and precise landmark-based localization, represents a refined, physiologically coherent evolution of AV junction ablation strategies. By directly targeting the compact node rather than the insulated His bundle, this method minimizes unnecessary myocardial damage, avoids abrupt disruption of His–Purkinje conduction, and preserves a stable and reliable junctional escape rhythm. The ability to identify nodal potentials with high-resolution signal filtering further enhances procedural confidence, allowing operators to deliver radiofrequency energy with unprecedented spatial accuracy. Beyond its acute efficacy, this approach has the potential to reduce proarrhythmic scar formation, limit the risk of sudden QRS modifications and QT dispersion, and improve long-term arrhythmic stability in vulnerable patient populations. Future studies are warranted to validate these findings, refine procedural endpoints, and standardize techniques that may ultimately redefine the therapeutic paradigm for patients requiring definitive AV nodal ablation.

## Data Availability

No new data were created or analyzed in this study.
